# Factors Associated with Inconsistent Condom Use among Men Who Have Sex with Men in Cambodia

**DOI:** 10.1371/journal.pone.0136114

**Published:** 2015-08-19

**Authors:** Siyan Yi, Sovannary Tuot, Pheak Chhoun, Khuondyla Pal, Khimuy Tith, Carinne Brody

**Affiliations:** 1 Research Center, KHANA, Phnom Penh, Cambodia; 2 Center for Global Health Research, Public Health Program, Touro University California, Vallejo, California, United States of America; University of Toronto, CANADA

## Abstract

**Background:**

Compared to the general population, men who have sex with men (MSM) are at greater risk for HIV and less understood due to their more hidden and stigmatized nature. Moreover, the discrepancy in findings in the literature merits further investigations in MSM populations from different cultures and settings. We therefore conducted this study to explore factors associated with inconsistent condom use among high-risk MSM in Cambodia.

**Methods:**

This cross-sectional study was conducted in 2014 among 367 MSM randomly selected from Battembang and Siem Reap using a two-stage cluster sampling method. A structured questionnaire was used for face-to-face interviews to collect information on characteristics of respondents, HIV testing history, self-perception of HIV risk, substance use, sexual behaviors, mental disorders, and HIV knowledge. Multivariable logistic regression analysis was performed to identify factors independently associated with inconsistent condom use.

**Results:**

On average, 62.3% of respondents reported that they always used condoms over the past three months. The rates varied with types of sexual partners; the proportion of respondents who reported always using condoms was 55.1%, 64.2%, 75.9%, 73.0%, 78.1%, and 70.3%, for sexual partners who were girlfriends, boyfriends, female sex workers, male sex workers, female clients, or male clients, respectively. After adjustment, inconsistent condom use was significantly associated with age of ≥25 (AOR = 1.77, 95% CI = 1.09–2.86), self-rated quality of life as good or very good (AOR = 4.37, 95% CI = 1.79–5.67), self-perception of higher HIV risk compared to the general population (AOR = 2.37, 95% CI = 1.35–4.17), illicit drug use in the past three months (AOR = 5.76, 95% CI = 1.65–10.09), and reported consistent lubricant use when selling anal sex to men in the past three months (AOR = 2.85, 95% CI = 1.07–8.12).

**Conclusions:**

We found risky sexual behaviors to be considerably high among MSM in this study, especially among those who used illicit drugs or were older than 25. HIV education and social marketing should be expanded and specifically designed for MSM to better educate on the increased risk of HIV with unprotected anal sex and illicit drug use as risk factors, and the importance of the use of both condoms and lubricant during anal intercourse.

## Introduction

With advances in HIV prevention and treatment, there is cause for optimism that control of the HIV epidemic is achievable in many high-burden countries [1,2]. In the developing world, the rates of new HIV infections has been declining, and access to care and treatment services have improved significantly. This is true even in resource-limited countries in sub-Saharan Africa [2]. However, HIV epidemiology has recently changed globally [3]. Recent reports have highlighted that, across both underdeveloped and developed countries, less progress has been made in reducing HIV prevalence and incidence among men who have sex with men (MSM) [4,5]. Particularly in low- and middle-income countries, MSM are at greater risk for HIV acquisition and transmission due to their more hidden and stigmatized nature [6,7], compounded by limited access to basic prevention materials such as condoms and lubricant [4]. Analyzing data from 82 peer-reviewed articles, 12 surveillance reports, and 38 country progress reports, Beyrer and colleagues found that the pooled HIV prevalence in MSM ranged from 3·0% in the Middle East and north Africa region to as high as 25·4% in the Caribbean [4].

In Cambodia, despite the encouraging declines in new HIV infections in the general population [8], HIV prevalence and sexual behaviors among MSM remains of great concerns [8,9]. The BROS Khmer Study conducted in 2010 found that the prevalence of HIV and sexually transmitted infections (STIs) among MSM in Cambodia were 2.2% and 51.5%, respectively [9]. Moreover, according to the recent Cambodia Behavioral Sentinel Surveillance (BSS 2013), 18.3% of MSM reported having paid for sex with men. Within that group, only 38.1% reported always using condoms, and 41.3% reported always using lubricant with male sex workers in the past month [10]. The survey also showed that 26.9% of MSM had sold sex to men in the past 12 months, and the rates of consistent condom and lubricant use with male clients in the past months was as low as 35.7% and 49.5%, respectively [10]. Similar to MSM in other settings, 66% of MSM in Cambodia in the BSS 2013 reported having sex with women, and 54% reported having sex with transgender individuals (TG) in the past 12 months [10]. The prevalence of consistent condom use when having sex with women and TG was 52.2% and 67.4%, respectively [10]. These findings clearly indicate that MSM in Cambodia are at much higher sexual risk compared to the general population.

Increased condom use as a means to reduce the number of new HIV infections among MSM is a public health priority, and a critical component in prevention programs globally [11–13]. Risk of HIV transmission in unprotected anal sex is approximately 18 times higher than unprotected vaginal sex [14–16], and 70% of the risk could be reduced by consistent condom use [12]. Identifying factors associated with condom use is thus very important for designing effective prevention programs for this key population.

Several factors associated with condom use have been identified among MSM in different settings, although no studies have been published on factors associated with condom use among MSM in Cambodia. In Asian countries, condom use was found to be associated with substance use [17–20], cohabiting with a steady male or female partner [20], and trust between partners [21]. Other studies in Asia have reported the associations between condom use and sexual behaviors among MSM such as having multiple sex partners [20], sexual positions [20], types of sex partners [22], frequency of receptive anal sex with regular partners [18], number of casual partners [18], and early anal sex debut [20]. Several important perception and attitudinal factors have also been found to be associated with condom use among Asian MSM, including positive attitudes towards condom use [21], perceived condom acceptability by their partners [17], perceived ability to control condom use in their relationships [21,23], self-efficacy in insisting on condom use [24], and perceived efficacy of condom use for HIV prevention [23].

Other studies outside of Asia have found a variety of different factors associated with condom use among MSM including younger age [25,26], substance use [25,27,28], history of forced sex [27], having both regular and casual partners [27], loneliness [29], social support in communities [30], poor HIV knowledge [26,27,30], perceived AIDS stigma [26], and a history of HIV testing [28].

The findings on the relationships between self-perception of HIV risk and condom use among MSM are mixed. A study in India found that self-perception of higher risk of acquiring HIV was associated with consistent condom use [18], while studies in Cote d'Ivoire [27] and China [23,24] found the contrary, that self-perception of high HIV risk was associated with inconsistent condom use among MSM. This discrepancy merits further investigations in MSM populations from different cultures and settings. Clearly, condom use among MSM is influenced by context-specific factors and without evidence on the factors associated with condom use among MSM in Cambodia, we will not be able to address the underlying needs of this population in the country. We therefore conducted this study to explore factors associated with condom use among high-risk MSM in Cambodia.

## Materials and Methods

### Ethical statement

Participation in this study was voluntary. In the process of obtaining their written informed consent, participants were made clear that they could refuse or discontinue their participation at any time and for any reason. To ensure the confidentiality, privacy of the respondents was protected by conducting the interviews at a private place, and personal identifiers were not used in the survey questionnaires and field notes. The National Ethics Committee for Health Research of the Ministry of Health, Cambodia approved the study protocol and materials (Reference no. 082NECHR).

### Study population and sampling

Data used for this analysis were collected as part of an impact evaluation of the Sustainable Action against HIV and AIDS in Communities (SAHACOM) project implemented by KHANA, the largest national NGO providing integrated HIV prevention, care, and support services at the community level in Cambodia. The survey was conducted in Battembang and Siem Reap provinces between April and May 2014. Details of this evaluation study have been published elsewhere [31–34].

In this survey, 394 MSM were randomly selected from venues and hotspots identified by community health workers. To select the study sample, a two-stage cluster sampling method was used, and communes in each province were used as the smallest unit for the sampling process. In Cambodia, communes are the third-level administrative divisions, and they are subdivisions of the districts. Communes can consist of as few as 3 or as many as 30 villages, depending on the population. Only communes with at least 20 MSM were included in the sampling frame. The probability proportional-to-size sampling method was then used to select the required number of MSM from each commune. A person would be included in this study if he was: (1) 18 years or older; (2) self-reported as an MSM; (3) sexually active in the past three months; (4) able to provide consent to participate in the study; and (5) available for a face-to-face interview on the day of the data collection. A selected MSM would be excluded from the study if he was mentally and/or physically too sick to attend the interview. In this study, 27 (6.9%) MSM who had not been sexually active in the past three months were excluded from the analyses.

### Questionnaire development and training

We first developed a structured questionnaire in English and then translated it into Khmer, the national language of Cambodia. The Khmer questionnaire was back-translated and pretested with a sample of 10 MSM in Phnom Penh to ensure that the wording and contents were culturally suitable and clearly understandable for the respondents. We also received comments from experts working on HIV in key populations in Cambodia, and the questionnaire was finalized based on their feedbacks and findings from the pilot study.

All research team members spent three days for training on data collection methods. The training focused on building familiarity with the study protocol and questionnaire, interview techniques, privacy assurance, and confidentiality. The training also addressed quality control strategies, such as rechecking and reviewing the questionnaires after administration and resolving issues that might arise during the fieldwork. Data collection team leaders were encouraged to perform regular reviews with data enumerators to review progress and communicate any issues occurring during the data collection.

### Variables and measurements

The survey questionnaire was developed by using standardized tools adapted from a previous study in the same population [35], the most recent Cambodia Demographic and Health Survey [36], as well as from other studies in Cambodia [37–40] to measure socioeconomic characteristics, exposure to HIV education, history of HIV counseling and testing, self-rated overall health and quality of life, self-perception of HIV risk compared to the general population, sexual behaviors, substance use, mental disorders, and HIV knowledge.

Socioeconomic characteristics included age, years of formal education completed, marital status, living situations, main occupation, average monthly income, and personal perception on their own sexual identity. Yes/no questions were used to assess risky sexual behaviors including sexual activities, as well as condom and lubricant use with different partners such as regular female partners, regular male partners, female sex workers, male sex workers, female clients, and male clients in the past three months. We also collected information on HIV-related education and sources of information they received in the past six months. We asked about alcohol and drug use in the past three months, age at the first use, and types of drugs used in the past three months.

HIV knowledge was assessed by using a 12-item scale adapted from a previous study [41]. The scale reflects information on HIV transmission, condom use, and HIV knowledge. The response options were ‘1 = no,’ ‘2 = yes,’ or ‘3 = don’t know.’ The total score of the scale was the sum of correct responses, with ‘don’t know’ responses scored as incorrect. Cronbach’s α among MSM in this study was 0.74. A short version of the General Health Questionnaire (GHQ-12) was used to measure mental disorders [42]. Each item of the scale was rated using four response options of “0 = less than usual,” “1 = no more than usual,” “2 = rather more than usual,” or “3 = much more than usual.” The scoring method ‘0-0-1-1’ has been suggested as it is believed to help eliminate biases resulted from respondents who tend to choose responses 0 and 3 or 1 and 2 [43]. To define lower and higher levels of mental disorders, the mean score for the whole population (3/4) was used as the cut-off as it provides a rough guide to the best threshold [44]. The Cronbach’s alpha among MSM in this study was 0.78.

### Data analyses

Double data entry was performed using EpiData version 3 (Odense, Denmark). Student’s *t*-test was used for continuous variables, and Chi-square test or Fisher’s exact test was used as appropriate for categorical variables to compare socioeconomic characteristics, self-rated overall health and quality of life, self-perception of HIV risk, sexual behaviors, substance use, HIV knowledge, and level of mental disorders (GHQ12) among MSM who reported always using condoms and those who reported not always using condoms in the past three months. To control for potential confounders, a multivariable logistic regression analysis was conducted. All variables significantly associated with condom use in bivariate analyses at a level of *p*< 0.05 were simultaneously included in the model. Variables with a *p*-value >0.05 were removed, and the model was refitted. The steps were repeated until all *p*-values of the remaining variables were <0.05 in the final model. Adjusted odds ratio (AOR) were obtained and presented with 95% confidence intervals (CI) and *p*-values. SPSS version 22 (IBM Corporation, New York, USA) was used for all statistical analyses.

## Results

### Condom and lubricant use with different partners

This study included 367 MSM with a mean age of 23.9 (SD = 5.2). Of total, 62.3% of the respondents reported always using condoms of in the past three months, and 86.0% reported using a condom at their last sexual intercourse. Slightly more than half (55.1%) reported always using condoms when having sex with girlfriends, and 64.2% reported always using condoms with boyfriends in the past three months. When asked about condom use with other partners, 75.9% of the respondents reported always using condoms when having sex with female sex workers, and 73.0% reported always using condoms when having sex with male sex workers. The rates of consistent condom use when selling sex to women and men were reported to be 78.1% and 70.3%, respectively. Regarding lubricant use, 80.8% of the respondents reported always using lubricant when having anal sex with boyfriends in the past three months. Of those who reported selling anal sex to men in the past three months, 64.1% reported always using lubricant.

### Socio-demographic characteristics

As shown in **Table 1**, MSM who reported not always using condoms were significantly older (mean age of 25.1 vs. 23.2) and more likely to have received HIV education through mass media in the past six months (66.1% vs. 52.0%) compared to those who reported always using condoms in the past three months. Regarding their perception of health and quality of life, MSM who reported not always using condoms were significantly more likely to rate their overall health (47.4% vs. 30.4%) and overall quality of life (44.5% vs. 19.6%) as good or very good and perceive their HIV risk as higher compared to the general population (45.3% vs. 36.0%). No significant difference was found in the comparison of the level of mental disorders using GHQ-12 score among the two groups.

**Table 1:** Comparisons of characteristics of MSM who reported always using condoms and MSM who reported not always using condoms.

Socio-demographic characteristics	Condom use in the past 3 months			
	Total (*n* = 367)	Always (*n* = 230)	Not always (*n* = 137)	*p*-value*
Mean age (in year)	23.9 ± 5.2	23.2 ± 4.5	25.1 ± 6.1	0.001
Gender identity				0.45
Men	207 (56.4)	124 (54.0)	83 (60.6)	
Women	79 (21.5)	53 (23.0)	26 (19.0)	
Other (including ‘both’)	81 (22.1)	53 (23.0)	28 (20.4)	
Marital status				0.13
Never married	329 (89.9)	212 (92.2)	117 (86.0)	
Married	29 (7.9)	15 (6.5)	14 (10.3)	
Divorced/separated/widowed	8 (2.2)	3 (1.3)	5 (3.7)	
Years of formal education completed	9.5 ± 3.2	9.4 ± 3.2	9.6 ± 3.2	0.67
Main occupations				0.91
Unemployed	15 (4.3)	10 (4.5)	5 (3.8)	
Students	92 (26.1)	61 (27.6)	31 (23.7)	
Farmer/laborer	57 (16.2)	34 (15.4)	23 (17.6)	
Self-employed	104 (29.5)	65 (29.4)	39 (29.8)	
Other	84 (23.9)	51 (23.1)	33 (25.2)	
Mean monthly income (in US$)	204 ± 611	758 ± 50	295 ± 25	0.38
Currently living with:				
Parents	257 (71.4)	170 (75.9)	87 (64.0)	
Relatives/siblings	36 (10.0)	21 (9.4)	15 (11.0)	
Spouse/sexual partner	30 (8.3)	15 (6.7)	15 (11.0)	
Friends/colleagues	24 (6.7)	13 (5.8)	11 (8.1)	
Other	13 (3.6)	5 (2.2)	8 (5.9)	
Mean years living in current city	19.6 ± 8.7	19.2 ± 8.0	20.2 ± 9.8	0.34
Received HIV education (past 6 months)	311 (85.0)	199 (86.9)	112 (81.8)	0.18
Sources of HIV education received in the past 6 months				
Media (TV/radio/newspaper)	178 (57.1)	104 (52.0)	74 (66.1)	0.02
Poster/billboard/booklet	83 (26.1)	57 (27.0)	29 (25.9)	0.83
Peer educator/outreach workers	282 (90.4)	180 (90.0)	102 (91.1)	0.76
Counseling at VCCT	15 (4.8)	10 (5.0)	5 (4.5)	0.83
Public health facilities	33 (10.6)	25 (12.5)	8 (7.1)	0.14
Other	30 (9.6)	20 (10.0)	10 (8.9)	0.76
Tested for HIV in the past 6 months	240 (77.7)	154 (78.6)	86 (76.8)	0.62
Self-rated overall health status				0.004
Good/very good	135 (36.8)	70 (30.4)	65 (47.4)	
Neither good nor poor	210 (57.2)	146 (63.5)	64 (46.7)	
Poor/very poor	22 (6.0)	14 (6.1)	8 (5.8)	
Self-rated overall quality of life				<0.001
Good/very good	106 (28.9)	45 (19.6)	61 (44.5)	
Neither good nor poor	243 (66.2)	173 (75.2)	70 (51.1)	
Poor/very poor	18 (4.9)	12 (5.2)	6 (4.4)	
Self-perception of HIV risk compared to the general population				0.006
Higher	132 (36.0)	70 (30.4)	62 (45.3)	
Same	64 (17.4)	39 (17.0)	25 (12.8)	
Lower	171 (46.6)	121 (52.6)	50 (36.5)	
Total score of General Health Questionnaire (GHQ12)				0.61
≤ 3	224 (62.4)	140 (61.4)	84 (64.1)	
≥ 4	135 (37.6)	88 (38.6)	47 (35.9)	

*Abbreviation*: *MSM*, *men who have sex with men; TV*, *television; VCCT*, *voluntary confidential counseling and testing*.

*Values are number (%) for categorical variables and mean ± SD for continuous variables*.

*Chi-square test or Fisher’s exact test was used for categorical variables and Student’s t-test was used for continuous variables.

### Alcohol and illicit drug use

**Table 2** compares alcohol and illicit drug use among MSM who reported not always using condoms and MSM who reported always using condoms in the past three months. MSM who reported not always using condoms were significantly more likely to be alcohol drinkers (91.2% vs. 80.9%), and their meant number of drunken days in the past month was also higher (6.2 ± 8.0 vs. 4.4 ± 5.8). In terms of illicit drugs, MSM who reported not always using condoms were significantly more likely to report using some kinds of illicit drugs in the past three months (8.8% vs. 1.7%).

**Table 2:** Comparisons of substance use among MSM who reported always using condoms and MSM who reported not always using condoms.

Substance use in the past 3 months	Condom use in the past 3 months			
	Total (*n* = 367)	Always (*n* = 230)	Not always (*n* = 137)	*p*-value*
Drunk at least a full glass of alcohol	322 (87.7)	186 (80.9)	125 (91.2)	0.04
Mean age at first alcohol intake	18.3 ± 4.3	18.1 ± 3.9	18.5 ± 4.8	0.38
Mean number of days getting drunk in past month	5.1 ± 6.8	4.4 ± 5.8	6.2 ± 8.0	0.02
Self-perception of level of alcohol drinking				0.29
Non-drinkers	28 (8.7)	19 (9.7)	9 (7.2)	
Social drinkers	287 (89.4)	175 (89.3)	112 (89.6)	
Heavy drinkers	6 (1.9)	2 (1.0)	4 (3.2)	
Used some kinds of illicit drugs	16 (4.4)	4 (1.7)	12 (8.8)	0.001
Mean age when trying illicit drugs for the first time	20.6 ± 7.4	16.8 ± 1.9	21.9 ± 8.2	0.24

*Abbreviations*: *MSM*, *men who have sex with men*.

*Values are number (%) for categorical variables and mean ± SD for continuous variables*.

*Chi-square test or Fisher’s exact test was used for categorical variables and Student’s t-test was used for continuous variables.

### Risky sexual behaviors

As shown in **Table 3**, mean number of sex partners in the past three months was significantly lower among MSM who reported not always using condoms than among MSM who reported always using condoms (4.7 ± 6.3 vs. 3.4 ± 3.8). However, MSM who reported not always using condoms were significantly more likely to report having sex with a girlfriend in the past three months (73.2% vs. 58.9%). Similarly, mean number of female sex workers they had sex with (3.3 ± 3.9 vs. 2.4 ± 1.8) and mean number of women they sold sex to (2.6 ± 2.1 vs. 1.6 ± 1.0) in the past three months were also significantly higher among MSM who reported not always using condoms. Interestingly, MSM who reported not always using condoms were significantly more likely to report always using lubricant when selling anal sex to men in the past three months (63.6% vs. 21.4%).

**Table 3:** Comparisons of sexual behaviors among MSM who reported always using condoms and MSM who reported not always using condoms.

Sexual behaviors in the past 3 months	Condom use in the past 3 months			
	Total (*n* = 367)	Always (*n* = 230)	Not always (*n* = 137)	*p*-value*
Mean number of sex partners	4.0 ± 5.5	4.7 ± 6.3	3.4 ± 3.8	0.03
Had sex with a girlfriend	118 (32.2)	66 (28.7)	52 (38.0)	0.04
Mean number of girlfriends you had sex with	1.7 ± 1.1	1.8 ± 1.1	1.6 ± 1.1	0.46
Had sex with boyfriends	205 (55.8)	126 (54.8)	77 (56.2)	0.40
Mean number of boyfriends you had sex with	2.4 ± 3.9	2.4 ± 3.8	2.1 ± 2.3	0.52
Always use lubricant when having anal sex with boyfriends	156 (80.8)	99 (82.5)	57 (78.1)	0.45
Had sex with FSWs	56 (14.6)	33 (14.4)	23 (16.8)	0.54
Mean number of FSWs you had sex with	3.0 ± 2.9	2.4 ± 1.8	3.3 ± 3.9	0.04
Had sex with MSWs	38 (9.9)	23 (10.0)	15 (10.9)	0.78
Mean number of MSWs you had sex with	2.0 ± 1.2	2.1 ± 1.3	1.8 ± 1.1	0.41
Sold sex to women	34 (8.9)	21 (9.1)	13 (9.5)	0.91
Mean number of female clients	1.9 ± 1.6	1.6 ± 1.0	2.6 ± 2.1	0.04
Sold sex to men	67 (17.4)	45 (19.6)	22 (16.1)	0.40
Mean number of male clients	2.3 ± 1.6	2.7 ± 1.7	1.9 ± 1.2	0.06
Always used lubricant when selling anal sex	41 (64.1)	9 (21.4)	14 (63.6)	0.001

*Abbreviations*: *FSWs*, *female sex workers; MSWs*, *male sex workers; MSM*, *men who have sex with men*.

*Values are number (%) for categorical variables and mean ± SD for continuous variables*.

*Chi-square test or Fisher’s exact test was used for categorical variables and Student’s t-test was used for continuous variables.

### HIV knowledge

**Table 4** shows that HIV knowledge among MSM in this study was generally good with the majority responding correctly to most of the HIV knowledge questions. However, it should be noted that a large gap in HIV knowledge was found in a question regarding if a person must have many different partners to get HIV. No significant difference was found in the comparisons of proportion of correct responses to all items of HIV knowledge among MSM who reported not always using condoms and MSM who reported always using condoms. The mean score of HIV knowledge was also not significantly different among these two comparison groups.

**Table 4:** Comparisons of correct responses to HIV knowledge items among MSM who reported always using condoms and MSM who reported not always using condoms.

HIV knowledge items	Condom use in the past 3 months			
	Total (*n* = 367)	Always (*n* = 137)	Not always (*n* = 230)	*p*-value*
Is AIDS spread by kissing? (No)^†^	354 (96.5)	220 (95.7)	134 (97.8)	0.39
Can a person get AIDS by sharing bathrooms with someone with HIV? (No)	347 (95.6)	216 (94.7)	131 (97.0)	0.43
Can men give HIV to women? (Yes)	359 (98.1)	227 (98.7)	132 (97.1)	0.43
Can women give HIV to men? (Yes)	365 (99.5)	228 (99.1)	137 (100)	0.53
Must a person have many different partners to get HIV? (No)	84 (22.9)	57 (24.7)	29 (21.1)	0.65
Can you get HIV by touching someone with HIV? (No)	348 (94.8)	217 (94.3)	131 (95.6)	0.60
Does washing after sex help protect against HIV? (No)	264 (71.9)	165 (71.7)	99 (72.3)	0.91
Is AIDS caused by spirits/supernatural forces? (No)	363 (98.9)	228 (99.1)	135 (98.5)	0.63
Can a pregnant woman give AIDS to her baby? (Yes)	270 (73.6)	176 (76.5)	94 (68.6)	0.10
Can a person get rid of AIDS by having sex with a virgin? (No)	244 (66.5)	161 (70.0)	83 (60.6)	0.06
Is HIV the virus that causes AIDS? (Yes)	313 (85.3)	197 (85.7)	116 (84.7)	0.80
Is there a cure for AIDS? (No)	330 (89.9)	210 (91.3)	120 (87.6)	0.25
Mean total HIV knowledge score	17.2 ± 1.3	17.2 ± 1.3	17.2 ± 1.3	0.99

*Abbreviations*: *MSM*, *men who have sex with men*.

^*†*^ *Correct responses are shown in parentheses*.

*Values are number (%) of respondents who answered correctly to the HIV knowledge items*.

*Chi-square test or Fisher’s exact test was used for comparisons of individual items, *and t-test was used comparison of the mean score*.

### Multivariable logistic regression analysis

**Table 5** shows factors associated with inconsistent condom use after controlling for other covariates in multivariable logistic regression model. After adjustment, inconsistent condom use remained significantly associated with the age group of ≥25 (AOR = 1.77, 95% CI = 1.09–2.86), self-rated quality of life as good or very good (AOR = 4.37, 95% CI = 1.79–5.67), self-perception of higher HIV risk compared to the general population (AOR = 2.37, 95% CI = 1.35–4.17), illicit drug use in the past three months (AOR = 5.76, 95% CI = 1.65–10.09), and consistent lubricant use when selling anal sex to men in the past three months (AOR = 2.85, 95% CI = 1.07–8.12).

**Table 5:** Factors associated with inconsistent condom use among MSM in multivariable logistic regression model.

Variables in the final model*	Inconsistent condom use in the past 3 months	
	AOR (95% CI)	*p*-value
Age group		
≤ 24	Reference	
≥ 25	1.77 (1.09–2.86)	0.02
Self-rated overall quality of life		
Neither good nor poor	Reference	
Poor/very poor	2.33 (0.62–8.72)	0.36
Good/very good	4.37 (1.79–5.67)	0.001
Perception of HIV risks compared to general population		
Same	Reference	
Lower	0.75 (0.39–1.47)	0.40
Higher	2.37 (1.35–4.17)	0.003
Illicit drug use in the past 3 months		
No	Reference	
Yes	5.76 (1.65–10.09)	0.006
Lubricant use when selling anal sex to men in the past 3 months		
Not always	Reference	
Always	2.85 (1.07–8.12)	0.03

*Abbreviations*: *AOR*, *adjusted odds ratio; CI*, *confidence interval; MSM*, *men who have sex with men*.

*Variables in the table were the ones that remained statistically significant after several steps of model fitting.

## Discussion

We found that sexual behaviors of MSM in this study such as having multiple commercial and non-commercial partners combined with inconsistent condom use may result in high risk of HIV and STI acquisition and transmission. Condom use rates across all types of relationship remained considerably low. Notably, almost half of MSM in this study perceived themselves at lower HIV risk compared to the general population. Since almost half of the respondents reported having female partners, MSM can be a potential bridge population for HIV transmission to other populations. Factors found to be associated with inconsistent condom use among these high-risk MSM included older age, self-rated quality of life as good or very good, self-perception of higher HIV risk compared to the general population, illicit drug use, and consistent use of lubricant when selling anal sex to men.

A number of health behavioral theories, including Protection Motivation Theory [45] and Health Belief Model [46], view risk perception as an important determinant of sexual behaviors. In this study, we found that MSM who perceived that they were at higher HIV risk were significantly more likely to report inconsistent condom use compared to those who perceived that they were at the same level of HIV risk compared to the general population. In this way, respondents correctly evaluated their risk based on their behaviors. Similar finding was reported in a study in Cote d'Ivoire, where MSM with self-perception of high HIV risk were significantly more likely to report inconsistent condom use during anal intercourse compared to MSM who perceived themselves at no HIV risk [27]. Studies in China also found that unprotected anal intercourse was associated with self-perception of high risk of contracting HIV [20,24] and perception of high HIV prevalence among MSM [23]. These findings may imply that, despite the awareness of their HIV risk, MSM continue to be involved in high-risk behaviors involving intimacy, closeness, and pleasure [20,47]. In contrast, self-perception of high HIV risk was associated with consistent condom use when having anal sex with paying and casual male partners in a study in India [18].

In this study, illicit drug users were significantly more likely to report inconsistent condom use compared to non-users. This finding is in line with reports from several studies that documented the relationship between the use of non-injecting drugs before and during sex and unprotected anal intercourse among MSM [17,28]. Furthermore, risky sexual behaviors, such as multiple sex partners and unprotected anal intercourse, has been linked to some specific types of illicit drugs including cocaine, methamphetamine, and poppers [48,49]. Previous studies suggest that the link between substance use and risky sexual behaviors might be explained by sensation-seeking behaviors, defined as a disposition characterized by the tendency to pursue novel, exciting, and optimal levels of stimulation [50]. Another possible explanation is that the intoxication with substances, such as methamphetamine, may have disorganizing effects on cognitive functions leading to poor decision-making on condom use [51]. This finding highlights the need for education on the risk related to the use of illicit drugs known to increase sexual arousal such as cocaine or methamphetamine and harm reduction programs tailored for MSM that may reduce the negative consequences of these behaviors.

We found that MSM in the age group of 25 or older were significantly more likely to report inconsistent condom use compared to younger MSM in the age group of 24 or younger. Studies in other Asian countries have reported similar findings that younger MSM are more likely to use condom consistently than older group [18,52]. However, younger MSM have often been found to engage more in unprotected anal intercourse in the United States [25] and Ghana [26]. Our finding may point out the predominant focus of HIV interventions on younger MSM population and limited access to prevention and care services among older MSM in Cambodia. It may also reflect ‘prevention fatigue’ among MSM in older group [53] that warrants tailored strategies to maintain preventive sexual behaviors and avoid relapsing of risky behaviors.

In general, HIV knowledge among MSM in this study was good, although room for improvement remains. However, levels of HIV knowledge was not related to levels of condom use. This finding is consistent with findings in a previous study in Ghana [30] and may reflect the lack of emphasis on same-sex sexual behaviors such as the high risk of anal intercourse in HIV education in Cambodia. It does not necessarily mean that HIV education is not important to promote condom use among MSM, but it suggests the need for HIV education materials that are specifically designed for MSM. Since anal sex is generally associated with same-sex sexual behaviors, it could be stigmatized and excluded from HIV education, representing a structural discriminatory barrier for HIV prevention [54]. In a study in Ghana, higher STI knowledge was associated with increased likelihood of condom use [30]. The importance of inclusion of specific knowledge items for MSM has been highlighted in previous studies, such as the information regarding the high risk of unprotected anal intercourse for HIV and STI transmission as well as the importance of lubricant use in anal intercourse [55,56]. Such information may change their perception on their risk exposure and in turn leads to the increased likelihood of consistent condom use. It is also important to integrate HIV and STI education, while HIV knowledge alone may not be sufficient to increase condom use.

Some limitations in this study should be noted. First, because of the limited sample size, we examined factors associated with overall condom use without specification on the types of partners or sexual positions, making the comparisons of our findings to those from previous studies difficult. Second, MSM in this study also included transgender women who might be different in characteristics and sexual behaviors. Recently, intervention programs for MSM and transgender women have been separated in Cambodia. Future studies should analyze the risk separately among MSM and transgender women. Third, as with any cross-sectional studies, we cannot make causal inferences, and we cannot determine whether the outcomes followed the risk factors in time or the risk factors resulted from the outcomes. Forth, we did not collect data on some important factors such as AIDS stigma and discrimination that have been found to be associated with sexual and health care seeking behaviors among MSM in previous studies. Regarding condom and lubricant, we collected only information on the separate use of them without specific questions about the concurrent use. Future studies should ask more specific questions on this important information. Fifth, our findings may be limited by the self-reported measures that may lead to inherent biases potential for both underreporting and over-reporting, although measures were taken to create conditions that encouraged valid responses from the respondents. Many topics we asked about were sensitive in nature including illegal activities such as drug use and sex work; information on these issues in particular were more likely to have been underreported. The final limitation concerns the representativeness of the study sample as we included only MSM from two provinces where the SAHACOM, a comprehensive community-based HIV project, has been implemented for MSM. Therefore, the levels of HIV risk and behaviors among MSM in this study may not represent the situation of general MSM population in other areas of Cambodia.

## Conclusions

Cambodia has made extensive progress in the implementation of structural community-based HIV interventions with service packages for key populations including MSM. However, our findings highlight several remaining issues that require particular attention. Risk for HIV and STI acquisition and transmission among MSM in this study are considerably high as many of them were involved in both heterosexual and homosexual relationships as well as multiple sexual partnerships, while levels of consistent condom use with different types of partners remained considerably low. Moreover, despite the risky behaviors, about half of respondents perceived themselves at lower HIV risk compared to the general population. HIV education and social marketing should be expanded and specifically designed for MSM addressing the increased risk of unprotected anal intercourse. Innovative methods of delivering more tailored risk reduction messages for a highly stigmatized population should be explored. There might be great misunderstanding about condom and lubricant use among MSM as we found that respondents who reported always using lubricant were less likely to report always using condoms. MSM may assume that lubricant can protect them from HIV and perceived less importance of consistent condom use. A message on the importance of the use of both condoms and lubricant during anal intercourse should be made clear in education sessions. Furthermore, our findings call for more attention on MSM older than 25 and integration of HIV risk reduction and substance use interventions.
